# ATP dependent NS3 helicase interaction with RNA: insights from molecular simulations

**DOI:** 10.1093/nar/gkv1378

**Published:** 2015-12-03

**Authors:** Andrea Pérez-Villa, Maria Darvas, Giovanni Bussi

**Affiliations:** Scuola Internazionale Superiore di Studi Avanzati, International School for Advanced Studies, 265, Via Bonomea, I-34136 Trieste, Italy

*Nucl. Acids Res*. 43 (18): 8725–8734. doi: 10.1093/nar/gkv872.

The ligand-protein and ligand-RNA electrostatic interactions reported in Table 2 were erroneously calculated taking into account only one atom from the ligand. As a consequence, the interactions were largely underestimated. The authors have recomputed the values including the whole ligand in the calculation. An up-to-date version of Table 2 is reported below. The values reported in the first three rows are not affected. The values reported in rows 4 and 5 are slightly affected. The values reported in the last two rows are significantly affected.

**Table 2.** Electrostatic interaction computed as Debye-Hückel energies (G^DH^). Protein* denotes Protein-RNA complex. Errors were computed from binning analysis (bin width: 80ns). Values lower that 0.05 kcal/mol are shown as “0.0”

	ΔG^DH^ (kcal/mol)	
RNA-Protein	Closed	Open
Apo	−4.9 ± 0.0	−3.7 ± 0.2
ADP·Mg^2+^	−5.0 ± 0.1	−3.9 ± 0.0
ATP·Mg^2+^	−5.5 ± 0.1	−4.7 ± 0.1
RNA-Ligand	Closed	Open
ADP·Mg^2+^	0.0 ± 0.1	0.0 ± 0.0
ATP·Mg^2+^	−0.2 ± 0.1	−0.3 ± 0.1
Ligand-Protein*	Closed	Open
ADP·Mg^2+^	−5.3 ± 0.1	−4.4 ± 0.1
ATP·Mg^2+^	−7.1 ± 0.0	−5.1 ± 0.1

As a consequence, a sentence in the third paragraph of the Section **Electrostatic interactions** should also be amended. Namely, sentence “Remarkably, ATP interacts more with the protein in its closed conformation, whereas ADP interacts more with the protein in its open conformation” should be changed to “Remarkably, ATP and ADP interact more with the protein in its closed conformation. The difference is more marked for ATP.”

The overall trends for the binding affinity of ATP are not changed. As a consequence this error does not affect any of the conclusions of the paper nor any of the issues commented in the **Discussion**.

The authors apologize for any inconvenience caused.

