# Supplementary material for: ATP dependent NS3 helicase interaction with RNA: insights from molecular simulations
Source: Nucleic Acids Res. 2015 Oct 10;43(18):8725–34. doi: 10.1093/nar/gkv872 (PMC4605317; doi:10.1093/nar/gkv872)
Supplement: SUPPLEMENTARY DATA [file supp_43_18_8725__index.html]

ATP dependent NS3 helicase interaction with RNA: insights from molecular simulations — SUPPLEMENTARY DATA 

# ATP dependent NS3 helicase interaction with RNA: insights from molecular simulations

## SUPPLEMENTARY DATA

- SUPPLEMENTARY DATA
- SUPPLEMENTARY DATA
