# Supplementary material for: ATP dependent NS3 helicase interaction with RNA: insights from molecular simulations
Source: Nucleic Acids Res. 2015 Oct 10;43(18):8725–34. doi: 10.1093/nar/gkv872 (PMC4605317; doi:10.1093/nar/gkv872)
Supplement: SUPPLEMENTARY DATA [file supp_gkv872_nar-02163-z-2015-File008.pdf]

ATP dependent NS3 helicase interaction with RNA: insights from molecular simulations

Andrea Pérez-Villa, Maria Darvas, and Giovanni Bussi

Supplementary Data

Content

**Table SD1:**	Number of atoms used in the set-up.
**Figure SD1:**	Ribbons representation for NS3h-ssRNA complex
**Table SD2:**	Subset of atoms selected to compute RMSD.
**Figure SD2:**	Projection of trajectories on (S,Z) variables for the first and second halves of the simulations.
**Figure SD3:**	RMSD plots for the long MD and for the control simulations.
**Table SD3:**	Distances between D1 and D2.
**Figure SD4:**	PCA of concatenated trajectories: Projection of the first two components.
**Table SD4-6:**	Average number of hydrogen bonds and their associated errors.
**Figure SD5:**	RNA binding cleft representation for closed and open conformations with ADP and apo.
**Figure SD6:**	Val432 representation in the ssRNA 5' terminus for closed and open with ATP.
**Figure SD7:**	Ligand binding site representation for closed and open conformations with ADP.
**Table SD7:**	Debye-Hückel interaction energies between RNA and protein residues.
**Table SD8:**	Enthalpy differences between open and closed conformations, for the first and second halves of the simulation.
**Figure SD8:**	Perfomed work vs simulation time obtained from Targeted MD.

Table SD1: Total number of atoms for every simulation.

**System**	Peptide	ssRNA	ligand	Mg^{2+}	Na^{+}	Cl^{-}	Water	Total atoms
**Apo**	6528	179	- - -	- - -	70	62	93174	100012
**ADP·Mg^{2+}**	6528	179	39	1	70	61	93174	100051
**ATP·Mg^{2+}**	6528	179	43	1	70	60	93174	100054

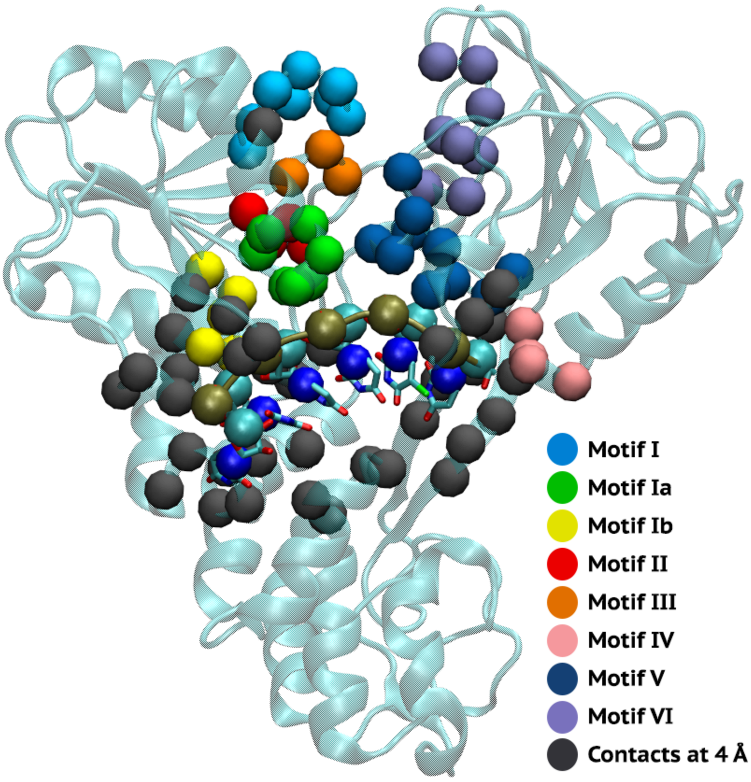

Figure SD1: Ribbons representation for NS3h-ssRNA complex. Nucleic acid is illustrated in sticks representation. Subset of atoms selected to compute RMSD are highlighted as solid spheres.

Table SD2: Subset of atoms selected to compute RMSD.

**Protein nodes**		Cα aminoacid
Motif I		204 to 211, Walker A
Motif Ia		230 to 235
Motif Ib		268 to 272
Motif II		290 to 293, DExH box
Motif III		322 to 324
Motif IV		369 to 372
Motif V		411 to 419
Motif VI		460 to 467, Arginine finger
Motif Y		Tyrosine 241
	D1	229, 253 to 256, 273 to 277 and 296 to 298
*around 4Å	D2	391 to 393, 410, 431 to 434 and 447 to 450
	D3	493, 497, 499 to 503, 551, 555, 556 and 558
**RNA nodes**		P, C4' and N1
Poly-uracil chain		U3 (5' terminus) to U8 (3' terminus)

*Protein contacts around 4 Å of RNA chain

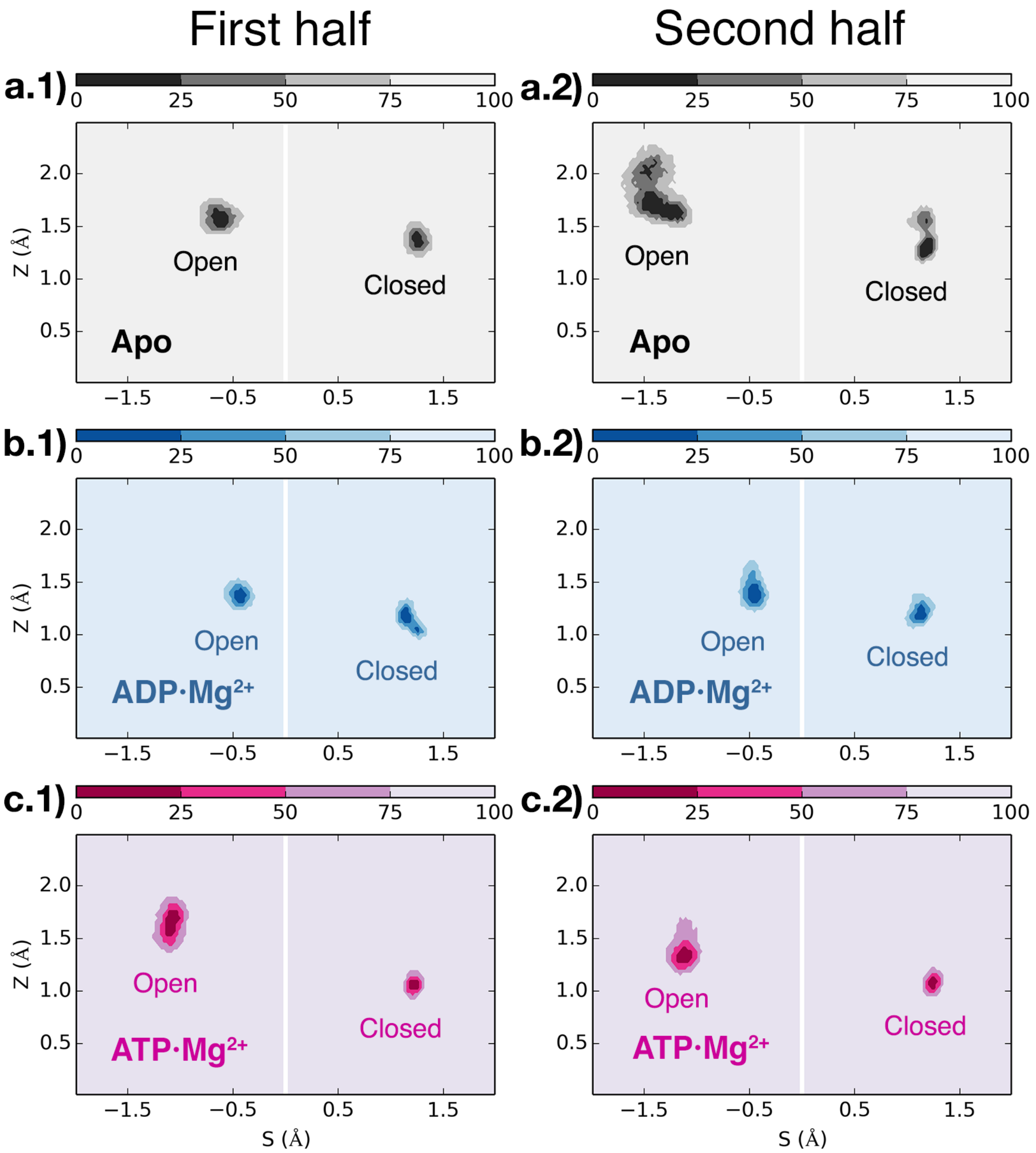

Figure SD2: Projection of trajectories on linear path variables (S,Z) computed for the first and second halves of the simulation. In the panel (a.2) it can be seen that the open-apo system visit a more open conformation during the second half of the simulation.

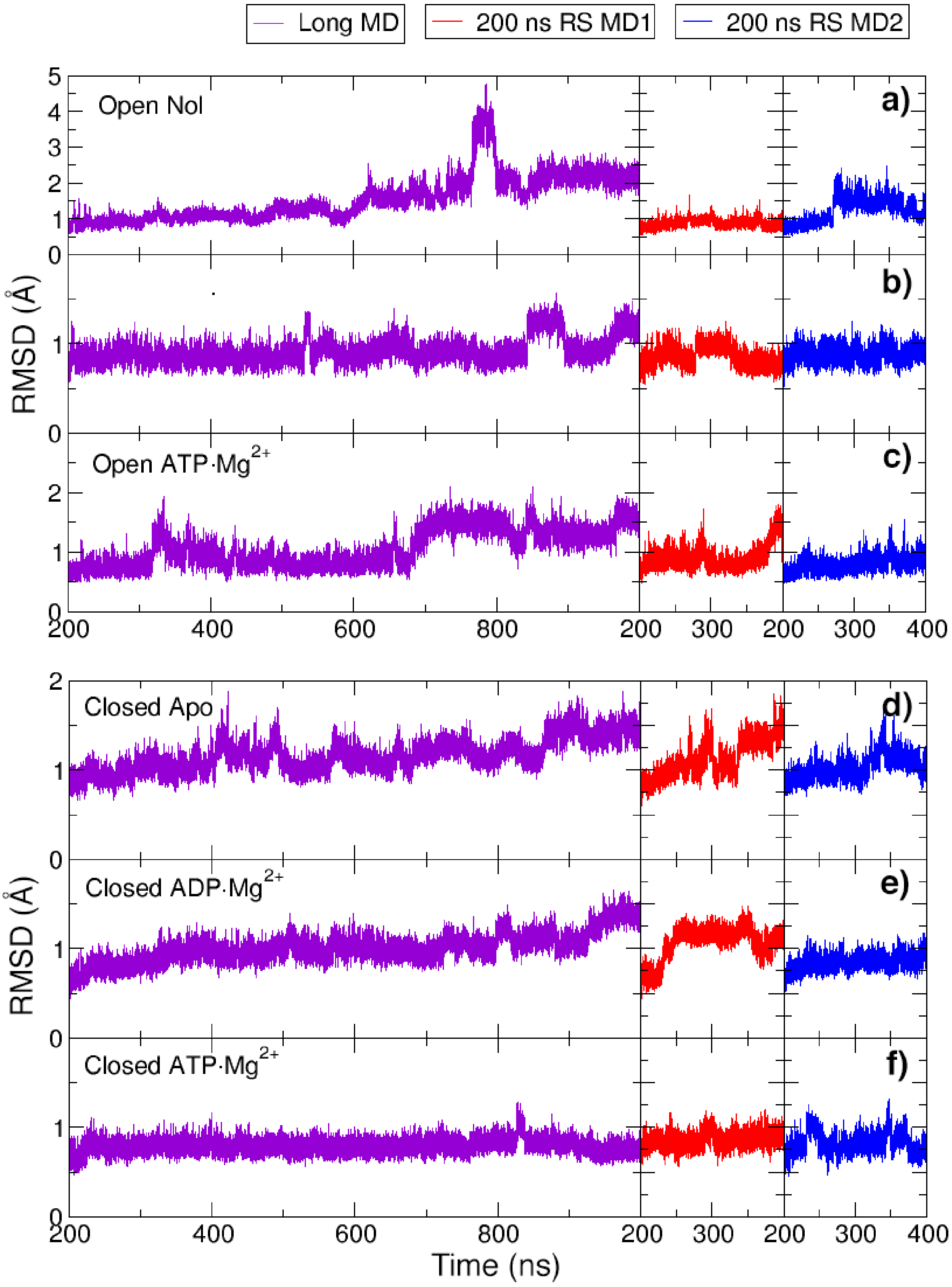

Figure SD3: RMSD plots for MD trajectories. Violet curves correspond to RMSD for long MD up to 1000 ns. Red and Blue curves are RMSD plots for the control MD simulations long 200 ns. Their starting structure corresponds to the configuration at 200 ns from the long MD simulation and their atomic velocities are initialized with a random seed. Least squares fit and RMSD calculations are done with the subset of atoms discussed in table SD2. A transitory jump is observed for the open apo system - panel (a) violet curve - due to a larger opening between D1 and D2. After that, the original intra-domain gap is recovered.

Table SD3: Gap between D1-D2 computed as the average distance between the centers of mass (COM) of D1 and D2. COM_{D1} and COM_{D2} are calculated considering all heavy atoms for residues 198-329 in D1 and 325-433 plus 450-483 in D2. Errors were calculated by block analysis, with a block width of 80 ns. Results for first and second halves of the trajectories are also shown, indicating that the open-apo system has a lower gap in the first part of the simulation, in agreement with the figure SD2. Errors lower that 0.05 Å are shown as "0.0".

	**COM_{D1}-COM_{D2} (Å)**	
**Full Trajectory**	**Closed**	**Open**
Apo	26.0 ± 0.0	28.9 ± 0.4
ADP·Mg^{2+}	25.8 ± 0.1	27.6 ± 0.0
ATP·Mg^{2+}	25.5 ± 0.0	28.5 ± 0.0
**First Half**	**Closed**	**Open**
Apo	26.1 ± 0.1	27.9 ± 0.0
ADP·Mg^{2+}	25.6 ± 0.1	27.6 ± 0.0
ATP·Mg^{2+}	25.6 ± 0.0	28.5 ± 0.0
**Second Half**	**Closed**	**Open**
Apo	25.9 ± 0.1	30.0 ± 0.5
ADP·Mg^{2+}	26.0 ± 0.1	27.6 ± 0.0
ATP·Mg^{2+}	25.5 ± 0.0	28.6 ± 0.1

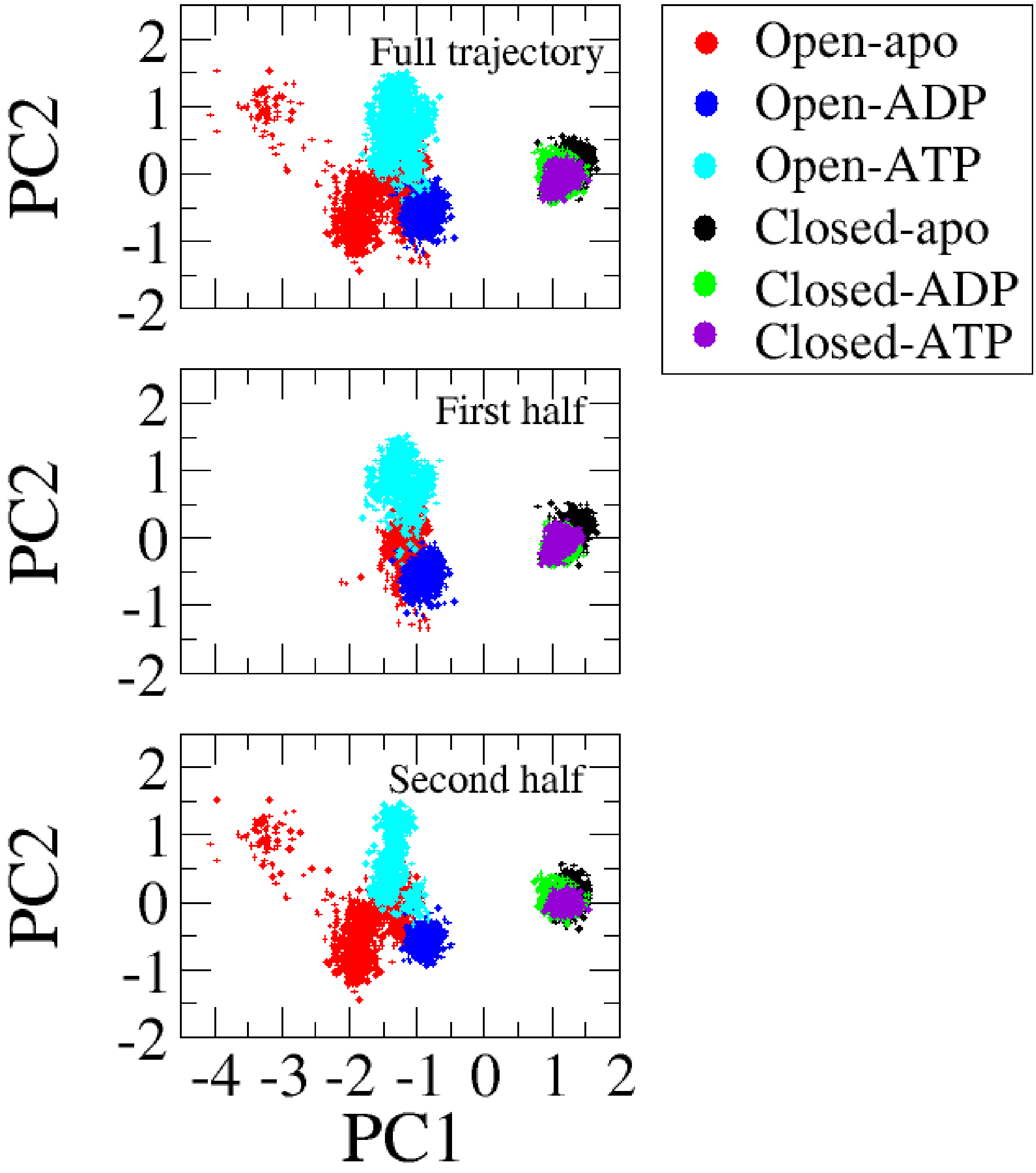

Figure SD4: Projection of the MD trajectories on the first two principal components. PCA is done on the trajectory obtained after concatenating all 6 simulations. Results for the whole trajectories are shown, as well as from first and second halves, as indicated. In agreement with what observed using the linearized path variables, all the open structures are more flexible and the open-apo explore a region which is very far from the closed structures. From this projection it is difficult to appreciate the decrease in the D1-D2 gap for the open-apo system (first half) and for the open-ADP system (whole trajectory). It is however clear that the open-apo system explore conformations with a large interdomain distance during the second part of the trajectory.

Table SD4: Average number of hydrogen bonds between solute groups for **NS3h-apo**. Errors computed from binning analysis (bin width: 80ns). Error bars lower that 0.05 Å are shown as "0.0".

**Ligand**	**First half of trajectory**		**Second half of trajectory**		**Full trajectory**		**Open crystal**
	Closed	Open	Closed	Open	Closed	Open	3O8C
D1-D2	5.5 ± 0.6	2.3 ± 0.2	5.5 ± 0.4	1.0 ± 0.2	5.5 ± 0.3	1.6 ± 0.3	0
D1-D3	2.5 ± 0.1	1.9 ± 0.1	2.5 ± 0.2	2.0 ± 0.3	2.5 ± 0.1	2.0 ± 0.1	0
D2-D3	7.5 ± 0.3	6.3 ± 0.2	7.8 ± 0.4	6.3 ± 0.1	7.6 ± 0.2	6.3 ± 0.1	7
D1-D1	99.3 ± 0.4	98.7 ± 0.4	97.7 ± 0.1	98.6 ± 0.6	98.5 ± 0.4	98.7 ± 0.3	90
D2-D2	114.5 ± 0.7	118.4 ± 0.2	112.8 ± 0.6	119.0 ± 0.8	113.6 ± 0.5	118.7 ± 0.4	110
D3-D3	104.6 ± 0.4	105.0 ± 0.2	104.8 ± 0.7	105.8 ± 0.5	104.7 ± 0.4	105.4 ± 0.3	101
D1-RNA	5.1 ± 0.3	3.8 ± 0.1	5.2 ± 0.1	4.3 ± 0.1	5.2 ± 0.1	4.1 ± 0.1	4
D3-RNA	7.0 ± 0.4	5.4 ± 0.3	7.7 ± 0.3	4.0 ± 0.4	7.4 ± 0.2	4.7 ± 0.3	4
D3-RNA	0.4 ± 0.1	3.7 ± 0.5	0.5 ± 0.1	4.2 ± 0.1	0.5 ± 0.0	4.0 ± 0.2	3
RNA-RNA	4.1 ± 0.2	3.7 ± 0.2	3.6 ± 0.1	3.0 ± 0.1	3.9 ± 0.1	3.3 ± 0.2	1
**Total**	350.5 ± 1.3	349.2 ± 0.8	348.1 ± 1.2	348.2 ± 1.3	349.4 ± 0.9	348.8 ± 0.8	320
ΔHB_{oc}	-1.3 ± 1.5		0.1 ± 1.7		-0.6 ± 1.2		

Table SD5: Average number of hydrogen bonds between solute groups for **NS3h-ADP**. Errors computed from binning analysis (bin width: 80ns). Error bars lower that 0.05 Å are shown as ”0.0”.

**Ligand**	**First half of trajectory**		**Second half of trajectory**		**Full trajectory**	
	Closed	Open	Closed	Open	Closed	Open
D1-D2	4.0 ± 0.3	2.3 ± 0.0	4.1 ± 0.5	2.3 ± 0.0	4.0 ± 0.3	2.3 ± 0.0
D1-D3	1.5 ± 0.1	1.7 ± 0.1	1.9 ± 0.3	1.8 ± 0.1	1.7 ± 0.1	1.8 ± 0.0
D2-D3	7.2 ± 0.2	6.9 ± 0.2	7.3 ± 0.4	6.3 ± 0.2	7.3 ± 0.2	6.6 ± 0.2
D1-D1	95.4 ± 0.7	96.4 ± 0.3	95.5 ± 0.6	96.9 ± 0.2	95.4 ± 0.4	96.6 ± 0.2
D2-D2	112.1 ± 1.1	115.8 ± 1.0	113.9 ± 1.1	116.4 ± 0.7	113.0 ± 0.7	116.1 ± 0.6
D3-D3	105.8 ± 0.8	103.9 ± 0.5	105.4 ± 0.6	104.5 ± 0.3	105.6 ± 0.5	104.2 ± 0.3
D1-ligand	6.9 ± 0.3	7.0 ± 0.1	8.9 ± 0.3	7.0 ± 0.1	7.9 ± 0.4	7.0 ± 0.0
D2-ligand	0.7 ± 0.2	2.7 ± 0.2	1.2 ± 0.3	2.4 ± 0.6	1.0 ± 0.2	2.6 ± 0.3
D3-ligand	0	0	0	0	0	0
Ligand-ligand	1.0 ± 0.0	0	1.0 ± 0.0	0	1.0 ± 0.0	0
RNA-ligand	0	0	0	0	0	0
D1-RNA	3.9 ± 0.1	3.8 ± 0.1	4.3 ± 0.3	4.0 ± 0.1	4.1 ± 0.2	3.9 ± 0.1
D3-RNA	7.4 ± 0.1	5.5 ± 0.0	7.4 ± 0.1	5.6 ± 0.1	7.4 ± 0.1	5.5 ± 0.1
D3-RNA	0.8 ± 0.1	2.1 ± 0.1	0.7 ± 0.1	1.9 ± 0.2	0.7 ± 0.1	2.0 ± 0.1
RNA-RNA	4.0 ± 0.1	5.1 ± 0.1	3.9 ± 0.2	4.9 ± 0.1	4.0 ± 0.1	5.0 ± 0.1
**Total**	350.7 ± 1.6	353.2 ± 1.2	355.5 ± 1.7	354 ± 1.1	353.1 ± 1.1	353.6 ± 0.8
ΔHB_{oc}	2.5 ± 2.0		-1.5 ± 2.0		0.5 ± 1.4	

Table SD6: Average number of hydrogen bonds between solute groups for **NS3h-ATP**. Errors computed from binning analysis (bin width: 80ns). Error bars lower that 0.05 Å are shown as "0.0".

**Ligand**	**First half of trajectory**		**Second half of trajectory**		**Full trajectory**		**Closed crystal**
	Closed	Open	Closed	Open	Closed	Open	3O8R
D1-D2	2.5 ± 0.2	0.4 ± 0.0	3.1 ± 0.4	0.4 ± 0.1	2.8 ± 0.2	0.4 ± 0.1	3
D1-D3	2.3 ± 0.1	1.8 ± 0.1	2.2 ± 0.1	1.8 ± 0.1	2.3 ± 0.0	1.8 ± 0.1	0
D2-D3	5.1 ± 0.2	6.7 ± 0.2	5.2 ± 0.0	6.1 ± 0.1	5.2 ± 0.1	6.4 ± 0.1	5
D1-D1	96.1 ± 0.2	95.3 ± 0.9	95.1 ± 0.3	94.3 ± 0.5	95.6 ± 0.2	94.8 ± 0.5	91
D2-D2	120.7 ± 0.3	113.5 ± 0.5	119.7 ± 0.9	115.7 ± 0.6	120.2 ± 0.4	114.6 ± 0.5	112
D3-D3	104.5 ± 0.3	105.0 ± 0.4	104.8 ± 0.2	103.6 ± 0.2	104.6 ± 0.2	104.3 ± 0.3	102
D1-ligand	7.7 ± 0.1	7.8 ± 0.1	7.7 ± 0.1	8.4 ± 0.3	7.7 ± 0.1	8.1 ± 0.2	6
D2-ligand	4.3 ± 0.1	1.0 ± 0.3	4.5 ± 0.1	0.3 ± 0.1	4.4 ± 0.1	0.6 ± 0.2	3
D3-ligand	0	0	0	0	0	0	0
Ligand-ligand	0.1 ± 0.0	1.0 ± 0.0	0.1 ± 0.0	1.0 ± 0.0	0.1 ± 0.0	1.0 ± 0.0	0
RNA-ligand	0	0	0	0	0	0	0
D1-RNA	4.2 ± 0.1	3.8 ± 0.0	4.5 ± 0.2	3.9 ± 0.0	4.4 ± 0.1	3.8 ± 0.0	3
D3-RNA	9.1 ± 0.2	5.4 ± 0.2	9.5 ± 0.1	6.0 ± 0.2	9.3 ± 0.1	5.7 ± 0.1	3
D3-RNA	1.0 ± 0.1	3.0 ± 0.1	1.0 ± 0.1	4.0 ± 0.2	1.0 ± 0.0	3.5 ± 0.2	0
RNA-RNA	4.3 ± 0.1	3.2 ± 0.1	4.5 ± 0.2	3.8 ± 0.3	4.4 ± 0.1	3.5 ± 0.2	1
**Total**	361.9 ± 0.6	347.9 ± 1.2	361.9 ± 1.1	349.3 ± 1.0	362 ± 0.6	348.5 ± 0.9	329
ΔHB_{oc}	-14.0 ± 1.4		-12.6 ± 1.5		-13.5 ± 1.1		

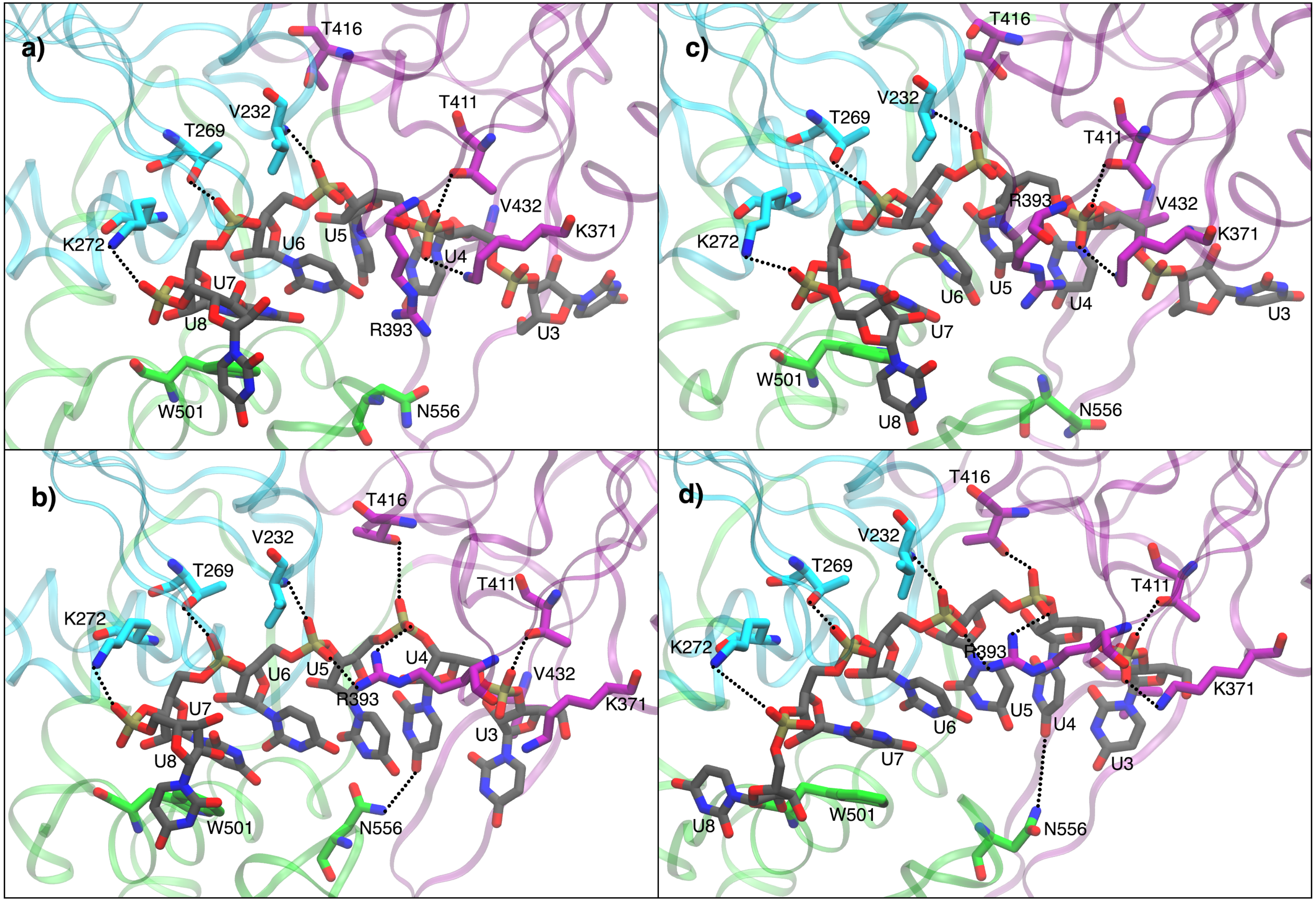

Figure SD5: Representation of the RNA binding cleft. Snapshots depicted correspond to the RMSD centroids of trajectories as obtained by the clustering algorithm(Daura et al. *Angew. Chem.* **1999**), using a cutoff radius of 1.25 Å . RNA chain and aminoacids interacting with RNA are highlighted in sticks representation. Residues from D1, D2 and D3 are depicted in cyan, purple and green respectively, RNA chain is shown in gray color. Snapshots are shown for closed-ADP (a), open-ADP (b), closed apo (c) and open apo (d) simulations.

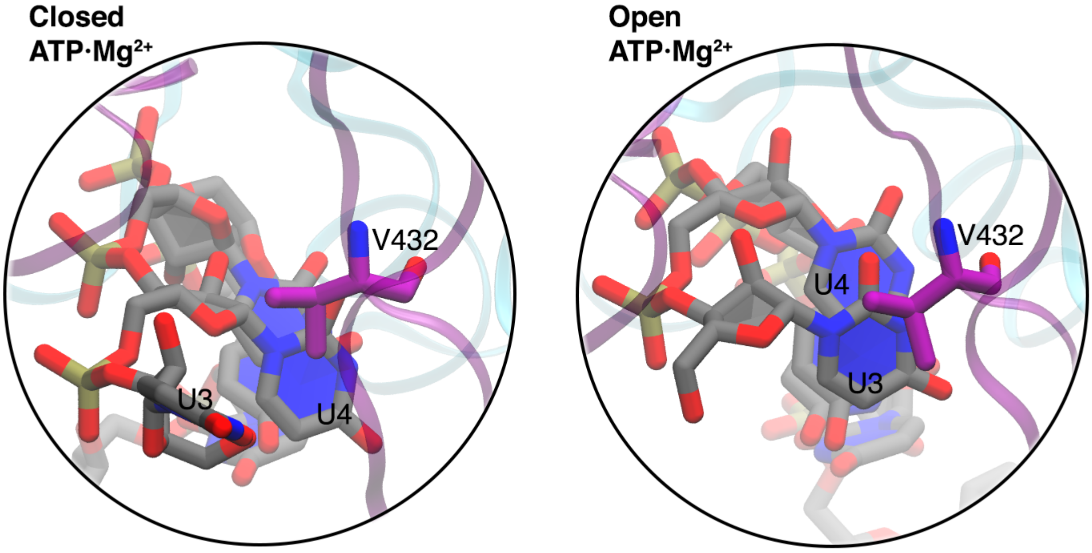

Figure SD6: Snapshot of RNA (gray) 5' terminus and Val432 (purple). The residues are depicted in stick representation.

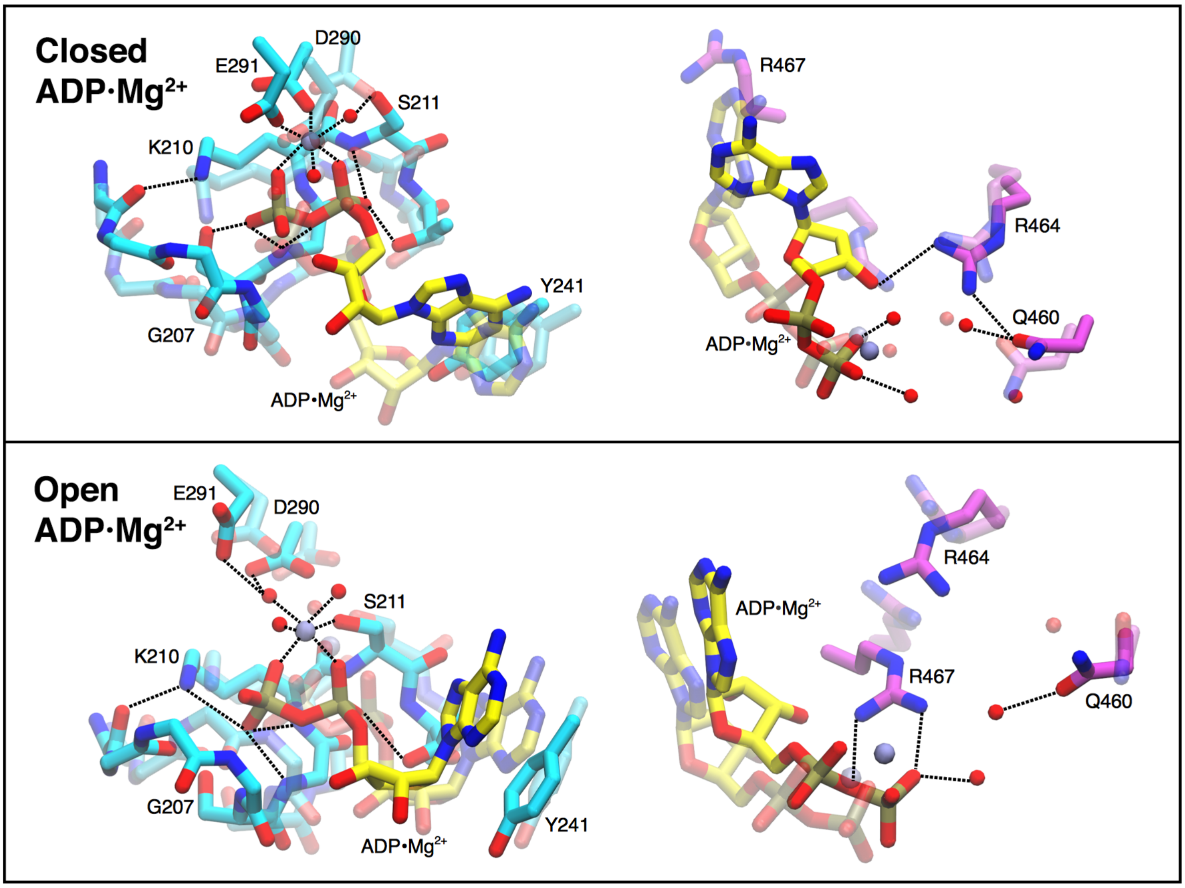

Figure SD7: Snapshots of the ATP/ADP binding site. Aminoacids from D1(cyan) and D2 (purple) and ligand (yellow) are shown as sticks, magnesium ion is shown as a lilac sphere. In the upper and lower panels are illustrated closed-ADP and open-ATP systems respectively. Transparent representation indicates the initial structure, solid color indicates the structure after 1μs of molecular dynamics. Coordination with Mg^{2+} cation and hydrogen bond interactions are shown with the black dashed line.

Table SD7: Debye-Hückel interaction energies (kcal/mol) between RNA and protein residues located in a range of 6 Å from the RNA chain. Errors are calculated from binning analysis (bin width: 80 ns) and are reported in parenthesis. Error bars lower than 0.05 kcal/mol are not shown.

Apo				ADP·Mg^{2+}				ATP·Mg^{2+}			
Closed		Open		Closed		Open		Closed		Open	
K371	-1.8	R393	-1.9	K371	-1.9	R393	-1.9	R393	-1.9	R393	-2.0
R393	-1.5(0.1)	K272	-1.0	R393	-1.4	K272	-1.0	K371	-1.8	K272	-1.0
K272	-1.0(0.1)	K371	-0.6(0.1)	K272	-1.0	K371	-0.8	K272	-0.9	K371	-0.9
K551	-0.4	K551	-0.5	K551	-0.4	K551	-0.4	K551	-0.4	K551	-0.5
K372	-0.4	S231	-0.2	K372	-0.4	T269	-0.2	K372	-0.4	T269	-0.2
T411	-0.2	T269	-0.2	T411	-0.2	S231	-0.2	T411	-0.2	V232	-0.2
T269	-0.2	V232	-0.2	T269	-0.2	T411	-0.2	T269	-0.2	S231	-0.2
S231	-0.2	G255	-0.2	S231	-0.2	V232	-0.2	V232	-0.2	T411	-0.2
G255	-0.2	T254	-0.2	G255	-0.2	G255	-0.2	G255	-0.2	G255	-0.2
V232	-0.2	T411	-0.1	S370	-0.2	T254	-0.1	S231	-0.2	K372	-0.2
S370	-0.2	K372	-0.1	V232	-0.2	Y392	-0.1	S370	-0.2	T254	-0.1
T254	-0.2	T416	-0.1	T254	-0.1	K372	-0.1	Y392	-0.1	Y392	-0.1
T298	-0.1	G271	-0.1	Y392	-0.1	A233	-0.1	T254	-0.1	T416	-0.1
Y392	-0.1	Y392	-0.1	H369	-0.1	N556	-0.1	H369	-0.1	A233	-0.1
G394	-0.1	A233	-0.1	T298	-0.1	G271	-0.1	Q434	-0.1	W501	-0.1
H369	-0.1	T298	-0.1	G394	-0.1	G417	-0.1	T298	-0.1	G271	-0.1
A233	-0.1	W501	-0.1	T448	-0.1	T298	-0.1	A233	-0.1	T298	-0.1
G271	-0.1	V256	-0.1	G271	-0.1	A275	-0.1	G271	-0.1	N556	-0.1
A275	-0.1	Y270	-0.1	A233	-0.1	V256	-0.1	A275	-0.1	V256	-0.1
V256	-0.1	A275	0	A275	-0.1	T416	0	N556	-0.1	A275	0
Q434	0	G417	0	V256	-0.1	Y270	0	V256	-0.1	Y270	0
Y270	0	G554	0	N556	0	G394	0	G394	0	G394	0
Y391	0	G394	0	Y270	0	L274	0	Y270	0	G417	0
L274	0	L274	0	L274	0	Q552	0	L274	0	H369	0
N556	0	N556	0	S297	0	S370	0	S297	0	L274	0
Y502	0	S297	0	Y391	0	T433	0	T448	0	S370	0
S297	0	T433	0	T450	0	T448	0	T450	0	A413	0
T448	0	S370	0	Y502	0	H369	0	Y502	0	S297	0
T450	0	T448	0	A413	0	S297	0	Q552	0	T433	0
W501	0	Y391	0	G417	0	Y502	0	T433	0	T448	0
A413	0	A497	0	Q552	0	A497	0	W501	0	T450	0
A497	0	H369	0	T416	0	A500	0	Y391	0	A497	0
A500	0	T450	0	W501	0	T450	0	A413	0	Q434	0
G417	0	Y502	0	A497	0	W501	0	A497	0	Y391	0
T416	0	A500	0	A500	0	Y391	0	A500	0	A500	0
T433	0	Q434	0	T433	0	A413	0	G417	0	Q552	0
T449	0	Q552	0	V432	0	Q434	0	V432	0	T449	0
V432	0	T449	0	T449	0	V432	0	T416	0	V432	0
Q552	0	A413	0	G554	0	G554	0	T449	0	Y502	0
G554	0	V432	0	P230	0.1	T449	0	G554	0	G554	0
P230	0.1	P230	0.1	Q434	0.1	P230	0.1	P230	0.1	P230	0
E503	0.2	E503	0.1	E503	0.2	E503	0.2	E503	0.2	E503	0.1
D412	0.5	D412	0.5	D555	0.5(0.1)	E493	0.5	D555	0.4	D555	0.4
D296	0.6	E493	0.6	D412	0.6	D412	0.5	D412	0.5	E493	0.5
E493	0.6	D555	0.7	D296	0.6	D296	0.6	D296	0.6	D412	0.5
D555	0.8(0.1)	D296	0.8	E493	0.6	D555	0.7	E493	0.6	D296	0.6

Table SD8: Enthalpy differences between open and closed conformations for the first and second halves of the simulation. The values are computed between systems with same number and kind of molecules. Error bars are computed from binning analysis (bin width: 80 ns).

**Ligand**	**ΔH_{oc} (kcal/mol)**	
	**First half**	**Second half**
**Apo**	-15.2 ± 3.5	-4.2 ± 3.8
**ADP·Mg^{2+}**	-28.8 ± 4.1	-21.8 ± 3.8
**ATP·Mg^{2+}**	-51.6 ± 7.6	-56.5 ± 4.6

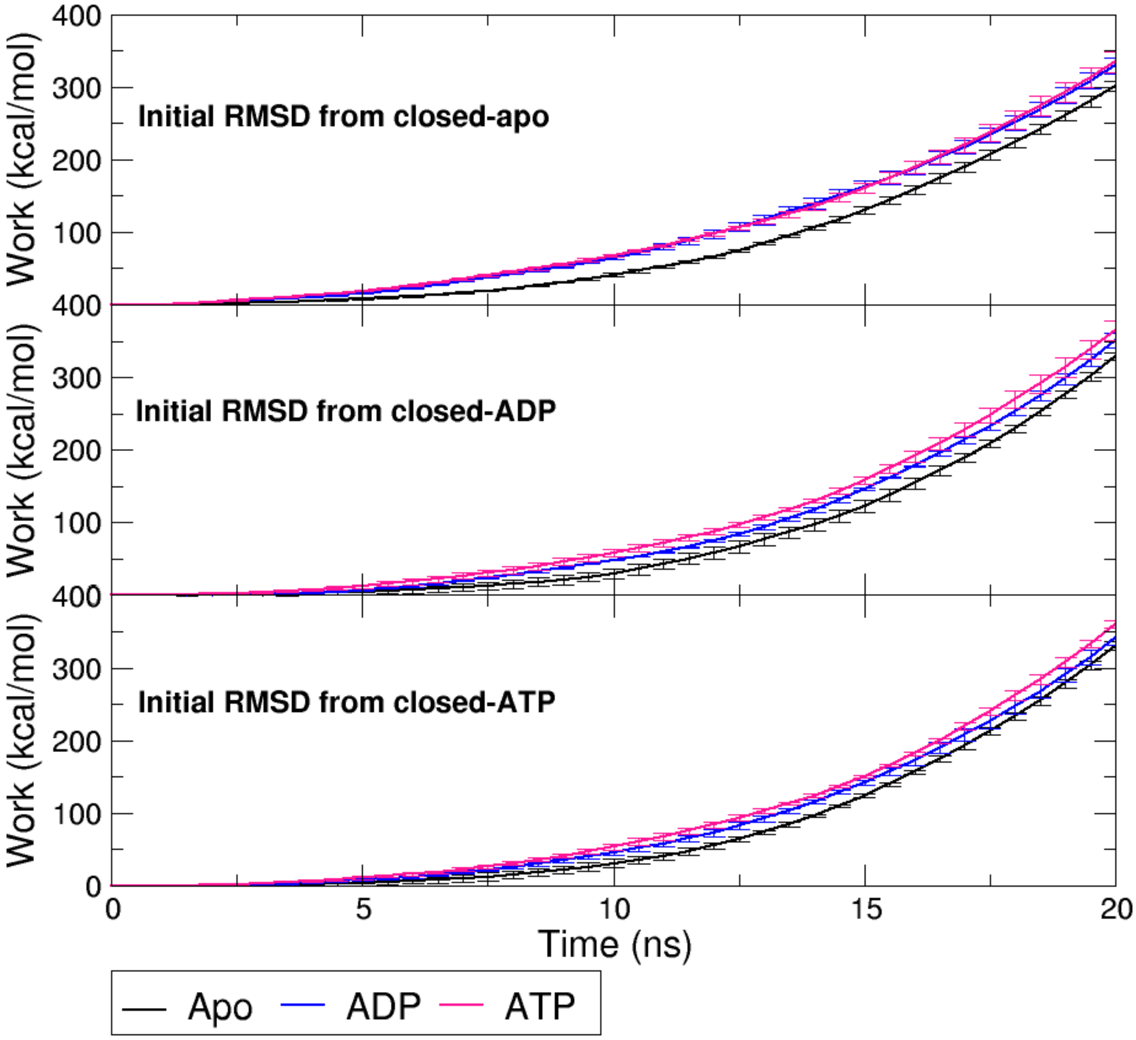

Figure SD8: Perfomed work vs simulation time from Targeted MD (TMD). Average work were obtained from 3 TMD simulations initialized with different seeds. Standard deviation of the three trajectories is shown as an error bar. The work required to open the apo structure is systematically lower (black line).
